# The Relative Efficacy of Chemically Diverse Small-Molecule Enzyme-Inhibitors Against Anticoagulant Activities of African Spitting Cobra (*Naja* Species) Venoms

**DOI:** 10.3389/fimmu.2021.752442

**Published:** 2021-10-07

**Authors:** Abhinandan Chowdhury, Matthew R. Lewin, Christina N. Zdenek, Rebecca Carter, Bryan G. Fry

**Affiliations:** ^1^ Venom Evolution Lab, School of Biological Science, University of Queensland, St. Lucia, QLD, Australia; ^2^ Department of Biochemistry & Microbiology, North South University, Dhaka, Bangladesh; ^3^ California Academy of Sciences, San Francisco, CA, United States; ^4^ Ophirex, Inc., Corte Madera, CA, United States

**Keywords:** venom, anticoagulant, Factor Xa, enzyme, inhibitor

## Abstract

African spitting cobras are unique among cobras for their potent anticoagulant venom activity arising from strong inhibition of Factor Xa. This anticoagulant effect is exerted by venom phospholipase A_2_ (Group I PLA_2_) toxins whose activity contributes to the lethality of these species. This anticoagulant toxicity is particularly problematic as it is not neutralized by current antivenoms. Previous work demonstrated this trait for *Naja mossambica, N. nigricincta*, *N. nigricollis*, and *N. pallida.* The present work builds upon previous research by testing across the full taxonomical range of African spitting cobras, demonstrating that *N. ashei*, *N. katiensis*, and *N. nubiae* are also potently anticoagulant through the inhibition of Factor Xa, and therefore the amplification of potent anticoagulant activity occurred at the base of the African spitting cobra radiation. Previous work demonstrated that the enzyme-inhibitor varespladib was able to neutralize this toxic action for *N. mossambica, N. nigricincta*, *N. nigricollis*, and *N. pallida* venoms. The current work demonstrates that varespladib was also able to neutralize *N. ashei*, *N. katiensis*, and *N. nubiae*. Thus varespladib is shown to have broad utility across the full range of African spitting cobras. In addition, we examined the cross-reactivity of the metalloprotease inhibitor prinomastat, which had been previously intriguingly indicated as being capable of neutralizing viperid venom PLA_2_ (Group II PLA_2_). In this study prinomastat inhibited the FXa-inhibiting PLA_2_ toxins of all the African spitting cobras at the same concentration at which it has been shown to inhibit metalloproteases, and thus was comparably effective in its cross-reactivity. In addition we showed that the metalloprotease-inhibitor marimastat was also able to cross-neutralize PLA_2_ but less effectively than prinomastat. Due to logistical (cold-chain requirement) and efficacy (cross-reactivity across snake species) limitations of traditional antivenoms, particularly in developing countries where snakebite is most common, these small molecule inhibitors (SMIs) might hold great promise as initial, field-based, treatments for snakebite envenoming as well as addressing fundamental limitations of antivenom in the clinical setting where certain toxin effects are unneutralized.

## 1 Introduction

In many tropical and subtropical nations, snake bite is a neglected public health issue, despite relatively cost-effective treatments available. Every year, approximately 5.4 million snake bites occur, resulting in around 81,410 – 137,880 deaths, along with many more amputations and permanent morbidity (1, 2). One of the most affected regions is Africa, where envenoming affects women, children, and farmers of poor rural communities primarily (3–5). In the Sub-Saharan regions of Africa, spitting cobras are responsible for a high percentage of envenomations, with the species responsible being *Naja ashei* (East Sub-Sahara), *N. katiensis* (Central and West Sub-Sahara), *N. mossambica* (Central, East, and South Sub-Sahara), *N. nigricincta* (Central and South Sub-Sahara), *N. nigricollis* (Central, East, South and West Sub-Sahara), *N. nubiae* (Central, East, North and West Sub-Sahara), *N. pallida* (East Sub-Sahara) (6–8).

African spitting cobras evolved around 15 million years ago, and represent one of the three lineages within the *Hemachatus/Naja* clade that have convergently evolved the spitting of venom trait (9–13). Venom cytotoxins cause severe pain and may damage the cornea, resulting in blindness if not washed thoroughly and treated early (14, 15). Clinical effects of envenoming include both local and systemic effects. Local effects include pain, blistering, swelling, and tissue death, while systemic effects include anticoagulant coagulopathy and flaccid paralysis (8, 14, 16–19). The diversity of effects are reflective of the complex composition of these venoms, which include three-finger toxins with cytotoxic and neurotoxic effects, anticoagulant phospholipase A_2_ toxins that inhibit Factor Xa and thrombin, and metalloproteases that cause edema and degrade fibrinogen (20–26).

While Factor Xa and thrombin inhibiting PLA_2_ toxins appear to be a basal trait in cobras, trait has only been strongly amplified in the African spitting cobras (26). While the mechanism of action for inhibiting thrombin has not been elucidated, the ability to inhibit FXa is well-described. Rather than inhibiting FXa active site directly, the toxins compete with Factor Va for binding to FXa at a site distinct from the enzymatic pocket (20, 26–29). These FXa-inhibiting toxins have been shown to unaffected by the SAVP polyvalent antivenom (which is made using the venoms of *Bitis arietans, Bitis gabonica, Dendroaspis angusticeps, Dendroaspis jamesoni, Dendroaspis polylepis, Haemachatus haemachatus, Naja nivea, Naja melanoleuca, Naja annulifera*, and *Naja mossambica*) but have been shown to be well neutralized by the PLA_2_ inhibitor varespladib (26). However, the study which demonstrated the efficacy of varespladib only used a single, high concentration of varespladib against a limited number of species. Thus, there is a knowledge gap regarding *ex vivo* efficacy of varespladib at varying concentrations and the broadness of its taxonomical utility in human blood. In addition, a recent study suggested that the metalloprotease inhibitor prinomastat cross reacts with venom PLA_2_ (30). That study, however, only included viperid venoms and thus the intriguing ability of prinomastat to neutralize anticoagulant activity of PLA_2_ in cobra venom was unexplored.

To fill this gap, our study set out to examine the concentration-dependent effects of varespladib across the full taxonomical range of African spitting cobras. We also ascertained the ability of prinomastat to cross-react with the anticoagulant PLA_2_ of these medically important snakes. In addition to undertaking the first anticoagulant toxicity testing for *N. ashei, N. katiensis*, and *N. nubiae* venoms, this work builds upon increasingly popular research into non-immunoglobulin treatments for snakebites using temperature-stable small molecule inhibitors (SMIs) that may improve snakebite first-aid in remote areas (20, 26, 30–34). Therefore, our results hopefully inform and streamline future platform testing while minimizing the need for *in vivo* animal testing. These approaches can be combined to expedite the development of improved treatments for snakebite and reduce dependence on animal models.

## 2 Materials and Methods

### 2.1 Stocks Preparation

#### 2.1.1 Venoms

Lyophilised venoms (*Naja ashei, N. katiensis, N. mossambica, N. nigricincta, N. nigricollis, N. nubiae*, and *N. pallida*) were purchased from a licensed biotechnology supplier, Latoxan (845 Avenue Pierre Brossolette, 26800 Portes-lès-Valence, France). These venoms were reconstituted to 4 mg/ml concentrated venom stock by adding 50% glycerol and storing at -20°C. The concentration was confirmed using a Thermo Fisher Scientific™ NanoDrop 2000 UV–Vis Spectrophotometer (Thermofisher, Sydney, Australia) set to 280 nm wavelength. All venom work was undertaken under the authority of UQ biosafety approval *#*IBC134BSBS2015.

#### 2.1.2 Plasma

Two bags of pooled 3.2% citrated recovered plasma (Label #A540020754341 & #A540020764777) were obtained from the Australian Red Cross (Research agreement #18-03QLD-09 and University of Queensland Human Ethics Committee Approval #2016000256). The two bags were defrosted and pooled together, aliquoted to 1 ml quantities, flash-frozen in liquid nitrogen, and stored at -80°C. For experimentation, aliquots were defrosted at 37°C in a Thermo Haake ARCTIC water bath. Defrosted plasma aliquots were replaced every hour for freshness. All plasma work was undertaken under the UQ biosafety approval #IBC134BSBS2015.

#### 2.1.3 Enzyme Inhibitors

Three small molecule inhibitors (SMI) were included to determine their efficacy against the included *Naja* venoms. Varespladib (varespladib:Na) was provided by Ophirex, Inc., (Corte Madera, CA, 94925, USA). Two metalloprotease inhibitors were purchased from Sigma-Aldrich: 1) prinomastat hydrochloride ((S)-2,2-Dimethyl-4- ((p-(4-pyridyloxy)phenyl) sulfonyl) -3- thio- from Sigma-Aldrich (catalogue# PZ0198) and 2) marimastat (2S,3R)- morpholinecarbohydroxamic acid hydrochloride) >95% (HPLC) N4-[(1S)-2,2-Dimethyl-1-[(methylamino)carbonyl] propyl]-N1,2- dihydroxy-3-(2-methylpropyl) butanediamide (catalogue # M2699). All the inhibitors arrived in powdered form, which were first dissolved in 10% dimethyl sulfoxide (DMSO) and further diluted using deionized water to form 10 mM concentration of metalloprotease inhibitor and varespladib solutions main stocks.

### 2.2 Assay Conditions

#### 2.2.1 Effects Upon Clotting Times of Plasma

##### 2.2.1.1 Coagulotoxicity Effects on Plasma and Fibrinogen

A STA-R Max^®^ (Stago, Asnières sur Seine, France) coagulation analyser was used to determine venom action (clotting time) on FXa inhibition. The method was modified from previously validated work from our lab (26). A working stock of 800 μg/ml was prepared by diluting 4 mg/ml venom stocks (50% glycerol) with Owren Koller buffer (OK buffer) (Stago catalogue #00360). The working stock was loaded into the analyser for running 8-point concentration curves, with 25 µl of the venom being serially diluted to: 114.3, 57.1, 38, 28.6, 22.9, 19, 16.3 and 14.3 µg/ml concentrations in the 175 µl incubation reaction (before addition of plasma), and 80, 40, 26.6, 20, 16, 13.3, 11.4 and 10 µg/ml concentrations in the 250 µL final reaction volume (subsequent to the addition of plasma). Subsequently added to the cuvette by the robot was 50 µl 0.025 M calcium (Stago catalogue # 00367), 25 µl of OK buffer, 50 µl of phospholipid (Stago catalogue #00597), and 25 µl of FXa (Stago catalogue # 00311). This was followed by a gentle shake-mix and then 2 minutes of incubation at 37°C. 75μl of plasma was then added and clotting time was measured immediately. To avoid venom degradation and maintain true replicates, fresh venom was loaded after each replicate of 8 dilutions. As a negative control test for FXa activity, 50% glycerol blank was used in place of venom.

##### 2.2.1.2 Enzyme-Inhibitor Efficacy

The efficacy of SMIs to neutralize toxic effects of venom upon plasma, the aforementioned 8-point concentration curves were repeated with the addition of SMIs in replace of 25 µl OK buffer. For the varespladib, the variant was prepared to a concentration 4 nM. For the metalloprotease inhibitors, the concentration used (2 mM) used was that has been shown previously to inhibit metalloproteases and also cross-react with PLA_2_ toxins (30, 35). For each inhibitor, there was an incubation reaction concentration of 0.0057 nM (varespladib) and 0.29 mM (prinomastat and marimastat), and a final reaction concentration (after addition of plasma) 0.4 nM (varespladib) and 0.2 mM (prinomastat and marimastat). As a negative control (FXa + Inhibitor) test for any effect of individual inhibitors on FXa activity, 50% glycerol blank was used in place of venom.

### 2.3 Statistical Analyses

To compare the area under the curve (AUC) of venom and venom + SMI, One-Way ANOVA, Multiple T-tests were made using GraphPad PRISM 8.1.1 (GraphPad Prism Inc., La Jolla, CA, USA). We calculated an X-fold magnitude of shift (formulae [(AUC of venom incubated with SMI/AUC of venom) - 1]) and later generating percentage (multiplying 100 with X-fold shift values) drop of clotting time was done using Excel. All raw data is available in the **Supplemental Material**.

## 3 Results and Discussion

We ascertained the ability of the PLA_2_-inhibitor varespladib and the cross-reactivity of the metalloprotease-inhibitors prinomastat and marimastat with the FXa-blocking anticoagulant PLA_2_ of African spitting cobras. Control tests with FXa added to plasma produced clotting in 12.1 +/- 0.2 seconds (positive control). Control tests of FXa incubated the inhibitors did not produce significantly different results, thereby showing the inhibitors did not affect the function of FXa: varespladib = 10.8 +/- 0.0; prinomastat = 12.4 +/- 0.5; marimastat = 11.2 +/-0.1.

**Figure 1:**
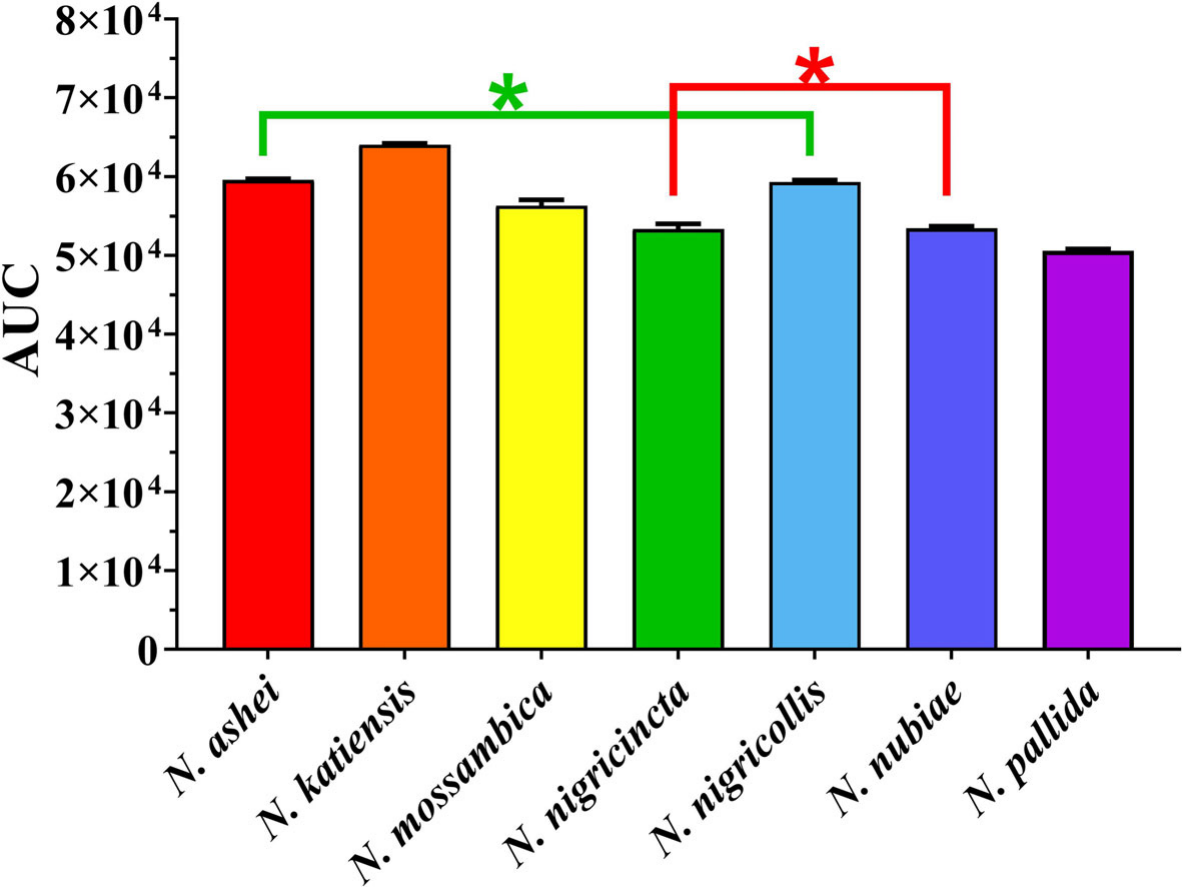
Y-axis showing area under the curve (AUC) of clotting times on human plasma produced through inhibition of FXa by African spitting *Naja* venoms (x-axis). Higher AUC indicated higher anticoagulant activity. *Represents no statistical significance AUC (*p* > 0.05) in Tukey’s multiple comparisons test at 95.00**%** confidence interval.

Consistent with a previous report (26), the *Naja* venoms showed a concentration-dependent potent inhibition of FXa (i.e. a slowing of the control clotting time), including the previously untested *N. ashei, N. katiensis*, and *N. nubiae* (**Figure 1**). Statistical analysis of AUC values revealed no significant difference only between *N. ashei* vs. *N. nigricollis* (*p* = 0.97) and *N. nigricincta* vs. *N. nubiae* (*p* = 0.99), while others had statistically significant differences in between them with *N. katiensis > Naja ashei* and *N. nigricollis >N. mossambica> N. nigricincta, N. nubiae* and *N. pallida* (higher AUC indicated greater anticoagulant activity). However, while there was a statistical difference in the potency between some species, it should be noted that all were extremely potent in their ability to inhibit FXa and the differences between them were not biologically significant in our opinion.

**Figure 2:**
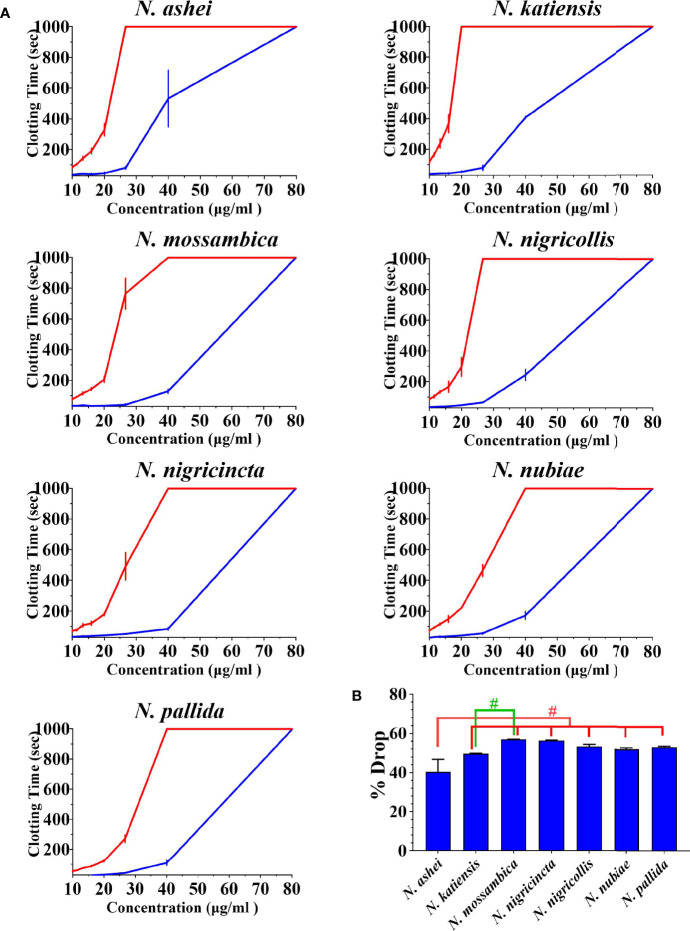
**(A)** 8-point concentration curves of venom vs varespladib. X-axis showing concentrations of venom in μg/ml and y-axis showing clotting times in seconds of human plasma with venom and relative inhibition efficacy of varespladib. Venom-induced clotting times shown as red curves, effect of venoms after pre-incubation with varespladib-Na as blue curves. Values are mean ± SD of N = 3 and shown as dots with error bars. Some error bars are too small to see. **(B)** Bar graphs of percentage drop of plasma clotting time due to induction varespladib-Na as blue bars. All Values are mean ± SD of N = 3. ^#^Represents statistical significance in percentage drops (*p* < 0.05) in Tukey’s multiple comparisons test at 95.00% confidence interval.

**Figure 3:**
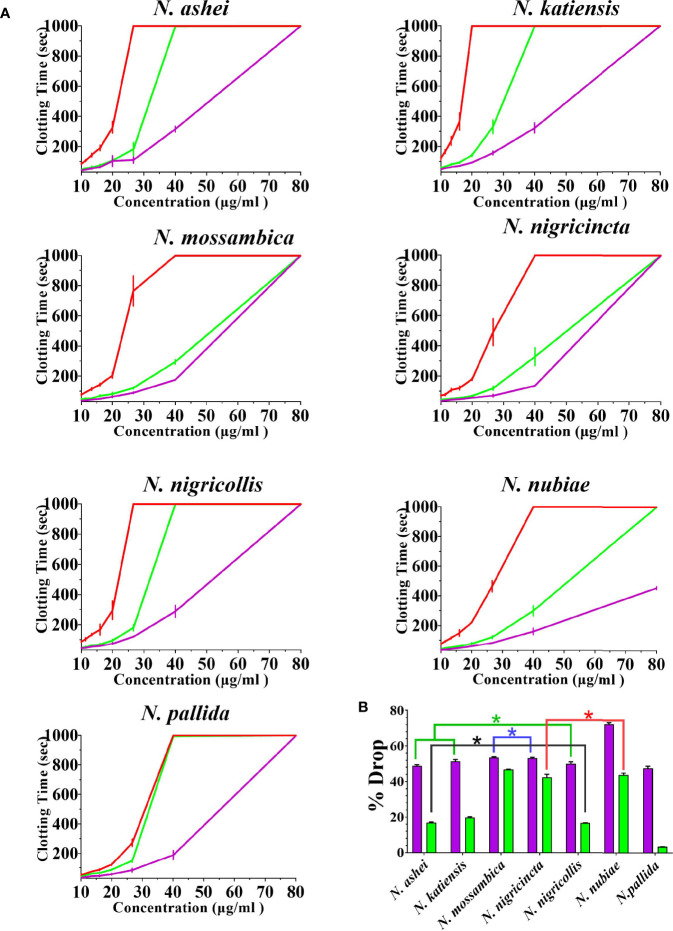
The ability of the two metalloprotease inhibitors to inhibit FXa-binding anticoagulant toxicity **(A)** 8-point concentration curves with venom-induced clotting times shown as red curves, effect of venoms after pre-incubation with prinomastat as purple curves, and effect of venoms after pre-incubation with marimastat as green curves. Values are mean ± SD of N = 3 and shown as dots with error bars. Some error bars are too small to see. **(B)** Bar graphs of percentage drop of plasma clotting time due to induction of prinomastat and marimastat, with prinomastat as purple bars, marimastat as green bars. Values are mean ± SD of N = 3. ^*^Represents no statistical significance (*p* > 0.005) in percentage drops in Tukey’s multiple comparisons test at 95.00% confidence interval.

Both varespladib and the metalloprotease inhibitors reduced the anticoagulant action on plasma produced by the *Naja* venoms (**Figure 2** and **Figure 3**). Despite being developed as specific metalloprotease inhibitors for the treatment of solid tumors, this study revealed that both prinomastat and marimastat cross-reacted with the PLA_2_-driven FXa-inhibition anticoagulant toxicity of all the venoms, with prinomastat being significantly more effective than marimastat (*p* < 0.05) for all species.

There were variations between each species regarding the action of an individual inhibitor. For example, varespladib-Na performed proportionally less effectively against *N. ashei* than the other species (*p* < 0.05, shown as # in **Figure 2**), ranging from 40.3 +/-6.4% drop (*N. ashei)* in plasma clotting times to 56.9 +/- 0.1% (*N. mossambica*) at the concentration used. However, while the difference is statistically significant relative to the other African spitting cobras, it was merely relatively less effective upon *N. ashei* than upon the other species which was still potent against, especially in light of the venom low concentration of varespladib needed to produce these drops. Prinomastat produced a drop in plasma clotting times ranging from a 72.5 +/- 0.8% drop for *N. nubiae* to a 49.9 +/- 1.2% drop for *N. nigricollis.* Marimastat also varied in efficacy, ranging from a 46.8 +/- 0.1% drop for *N*. *mossambica* to 3.4 +/- 0.1% for *N. pallida.* It must be noted that the prinomastat and marimastat concentrations were much higher than that of varespladib. These results are all strongly suggestive of the ability to recover normal clotting parameters but these results must be confirmed with *in vivo* studies and regarded as preliminary until then.

For all the inhibitors, the relative inhibition was not significantly inversely correlated to the venom anticoagulant potency (Pearson’s product-moment correlation test in R Studio values were: varespladib-Na cor = -0.45, p = 0.31; prinomastat cor = -0.28, p = 0.55; and marimastat cor = -0.17, p = 0.72). This suggests that the relative difference in efficacy between species was not due venom potency but instead due to differences in toxin surface biochemistry affecting the inhibitor’s ability to neutralize the toxin. A similar dynamic had been previously noted with a different inhibitor (4-benzenesulfonyl fluoride hydrochloride (AEBSF)) acting upon a different toxin type (kallikrein type serine protease) in the venoms of a different genus of snake (viperid genus *Trimeresurus*) (36). Such variations in underlying biochemistry impacting upon inhibitory actions is not uncommon. In the case of *Trimeresurus*, that study noted that the same species which were poorly neutralized by AEBSF, were also the ones which were poorly neutralized by antivenom. Thus suggesting that the same hypervariable sites were responsible for the two divergent effects. In other genera, significant variations in underlying biochemistry such as variable cofactor-dependency for clotting factor activation by diverse elapid and viperid snake venoms, impacts antivenom efficacy (30, 35, 37–40). Such variations underscore the fundamental principle that the dynamic diversification of snake venoms may have catastrophic effects on clinical treatment options (41, 42). Thus, the influences of different amino acid sequence and composition between toxins at the inhibitor binding site is a rich area of for future structure-function research.

This study is congruent with prior work, demonstrating the efficacy of varespladib against a wide range of snake venom PLA_2_-driven pathophysiological actions (20, 26, 30, 32–34, 43–48), thereby underscoring the potential utility of this compound as a first-aid option. For instance, field treatment options such as varespladib could stabilize patients in remote locations while being transported to hospital, and or serve as the only treatment option for some snake species such as the potent FXa-inhibiting anticoagulant effect of the African spitting cobra venoms included in this study (18, 24, 26, 49–51). An important caveat must be noted that these are *in vitro* results; as such, animal studies and human clinical trials must be conducted before recommendations for clinical use can be formally given. Regardless, evidence continues to build that varespladib shows tremendous promise as a temperature stable field treatment option for snakebite.

The cross-reactivity on PLA_2_ of the metalloprotease inhibitors prinomastat and marimastat was extremely intriguing. Particularly prinomastat showed strong cross-reactivity with the PLA_2_ (viperid venom Group II PLA2) toxicity at the same concentration (as in this study) at which it has been previously shown to be effective in neutralizing Factor X activating procoagulant SVMP (30, 35). Thus, prinomastat may be a broad-spectrum inhibitor of metalloproteases and PLA_2_ toxins at a single concentration dose. Congruent with the lower efficacy of marimastat noted in this study, that prior study demonstrated that marimastat was not able to neutralize the viperid venom PLA_2_ action at the concentration tested (30). Future work should include structural studies of the PLA_2_ toxins complexed with these metalloprotease inhibitors to ascertain the relative binding sites and thus how inhibition is accomplished. This is particularly intriguing in the case of the FXa-inhibiting toxins present in the venoms studied here as the inhibition is due to a non-enzymatic action of the toxin (27, 29). The two inhibitors differ significantly in their biochemistry: marimastat (MW 331.4 daltons) is simple, soluble aminopeptide; while prinoamstat (MW 423.5 daltons) is aromatic, sulfurous, alkaloid components. The influence of hydrophic groups may be a significant influence in relative effect, as the hydrophobic groups on varespladib sit in the groove of the PLA_2_ active site.

In conclusion, this study had multiple outcomes that improve our understanding of venoms, new and important avenues for enquiry and potential venom treatment options. 1) Through the first examination of the effects of *N. ashei, N. katiensis*, and *N. nubiae* venoms upon blood coagulation, it demonstrated that the ability of the venoms to strongly inhibit FXa is a trait that was amplified at the base of the African spitting cobra radiation. This finding contributes to our understanding of the evolutionary history of these fascinating snakes and provides supporting evidence for hypothesis that anticoagulant toxins play a key role in the defensive arsenal of African spitting cobras (26, 52). Whereby inhibition of clotting would promote the spread of destructive cytotoxins as vascular leakage as a consequence of the tissue damage, this would facilitate the local tissue damage to radiate outwards, leading to tremendous local damage characteristically evident in aftermath of envenomation African spitting cobras (53). 2) This study provided further data supporting the broad-spectrum efficacy of varespladib to inhibit PLA_2_-driven pathophysiological effects. 3) We demonstrated that the metalloprotease inhibitor prinomastat can surprisingly also inhibit PLA_2_ toxicity (at similar concentrations for both inhibition activities), thereby suggesting that this inhibitor may be a broad-spectrum neutralizer of multiple venom effects. Thus, not only did this study contribute to our understanding of the evolutionary biology of African spitting cobras, but it also provides additional supporting evidence for the continued development of SMIs to treat snakebite envenoming.

Future work should investigate the ability of prinomastat to inhibit other types of venom PLA_2_ toxins, such as the potent inhibition of the prothrombinase complex by the *Pseudechis* genus of Australian elapid snakes Group I PLA_2_ (33). Moreover examination of the site of action of prinomastat in blocking the PLA_2_ activity can be point of focus to expand knowledge on the fascinating cross-reactivity demonstrated against elapid venom Group I PLA_2_ here which corroborated a prior study (30).

## Data Availability Statement

The original contributions presented in the study are included in the article/**Supplementary Material**. Further inquiries can be directed to the corresponding author.

## Author Contributions

Study concept and design: AC, ML, RC, and BF. Experimental work: AC and BF. Manuscript first draft: AC and BF. Manuscript editing and revision: AC, ML, CZ, RC, and BF. All authors contributed to the article and approved the submitted version.

## Funding

BF was funded by Australian Research Council Discovery Project DP190100304 and AC was the recipient of a PhD Scholarship from the University of Queensland. ML and RC have shares and salary support from Ophirex, Inc., a Public Benefit Corporation and are funded in part by US Defense Health Agency contract W81XWH19C0082.

## Conflict of Interest

Authors ML and RC are employed by the company Ophirex. However, the company had no input in experimental design or reviewing of results before publication.

The remaining authors declare that the research was conducted in the absence of any commercial or financial relationships that could be construed as a potential conflict of interest.

The reviewer MBV declared a past co-authorship with the authors CZ and BF to the handling Editor.

## Publisher’s Note

All claims expressed in this article are solely those of the authors and do not necessarily represent those of their affiliated organizations, or those of the publisher, the editors and the reviewers. Any product that may be evaluated in this article, or claim that may be made by its manufacturer, is not guaranteed or endorsed by the publisher.
